# Transcription Factors That Govern Development and Disease: An Achilles Heel in Cancer

**DOI:** 10.3390/genes10100794

**Published:** 2019-10-12

**Authors:** Dhananjay Huilgol, Prabhadevi Venkataramani, Saikat Nandi, Sonali Bhattacharjee

**Affiliations:** Bungtown Road, Cold Spring Harbor Laboratory, Cold Spring Harbor, New York, NY 11724, USA; dhuilgol@cshl.edu (D.H.); venkatar@cshl.edu (P.V.)

**Keywords:** transcription factors, embryonic development, evolution, cancer, clinical trials, high mobility group box (HMG), basic helix loop helix (bHLH), paired box (Pax), GATA

## Abstract

Development requires the careful orchestration of several biological events in order to create any structure and, eventually, to build an entire organism. On the other hand, the fate transformation of terminally differentiated cells is a consequence of erroneous development, and ultimately leads to cancer. In this review, we elaborate how development and cancer share several biological processes, including molecular controls. Transcription factors (TF) are at the helm of both these processes, among many others, and are evolutionarily conserved, ranging from yeast to humans. Here, we discuss four families of TFs that play a pivotal role and have been studied extensively in both embryonic development and cancer—high mobility group box (HMG), GATA, paired box (PAX) and basic helix-loop-helix (bHLH) in the context of their role in development, cancer, and their conservation across several species. Finally, we review TFs as possible therapeutic targets for cancer and reflect on the importance of natural resistance against cancer in certain organisms, yielding knowledge regarding TF function and cancer biology.

## 1. Introduction

### 1.1. Embryonic Development and Cancer: Two Sides of the Same Coin 

Embryonic development involves a mass of cells achieving specific cell identities depending on morphogen gradients and the activation of transcription factors (TFs). These genetic changes propel ‘stem cells’ to form terminally differentiated cell types. In cancer, a terminally differentiated cell undergoes dedifferentiation to a stem cell, following which it assumes a new differentiated identity [1]. Interestingly, the cellular and molecular mechanisms are also quite conserved since tumorigenesis is caused by the reactivation of repressed genes [1]. If development is orderliness and regulation, cancer is deregulation. While in cancer, the accumulation of mutations in the genome leads to uncontrolled proliferation and the misdirected establishment of cell identity, embryonic development involves stem cell proliferation, fate specification, and migration must be streamlined in order to ‘assemble’ an organism. Studying embryonic tumors provides a case study as to how embryonic development can progress to cancer. Some examples include retinoblastoma (Rb), neuroblastoma, and nephroblastoma. An error in stem cell differentiation leads to each of these cancers. RB1, a tumor suppressor and well-known cell cycle regulator, has mutations in Rb [2]. However, a significant number of carcinomas are difficult to study since they are embedded amid a large population of differentiated cells [3]. 

Interestingly, a number of regulators of gene expression are also used as markers for cancer detection. Octamer binding transcription factor 4 (Oct4) is expressed in pluripotent stem cells and is necessary for controlling their pluripotency and self-renewal from gastrulation [4]. Intriguingly, it is also an established marker of tumor initiating cells (TIC) and embryonic carcinoma cells [5,6,7,8,9]. Oct4 plays an important role in the initiation and progression of cancer. The overexpression of Oct4, along with Sox2 and Nanog, has been shown to cause the dedifferentiation of glioblastoma multiforme cell lines into induced glioma stem cells [10,11]. In addition, OCT4 overexpression in human metastatic melanomas causes a loss of melanoma markers and increases cell migration [12,13]. RNAi mediated knockdown of Oct4 leads to reduced TIC phenotypes [13]. In lung adenocarcinoma, *Oct4* along with *Nanog* knockdown reversed the epithelial-to-mesenchymal transition (EMT) and inhibited tumorigenesis and metastasis [14]. 

**Figure 1:**
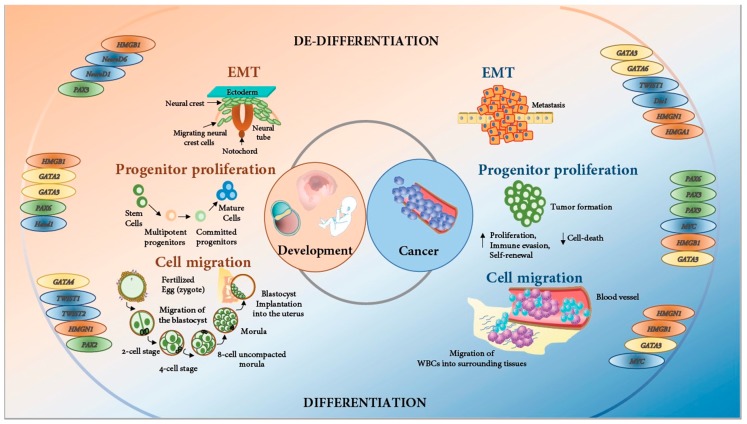
Development and Cancer: Two sides of the same coin. Schematic representation of parallel processes in development and cancer. Three examples of these processes have been outlined: i) EMT, ii) progenitor proliferation and, iii) cell migration. In the context of development, EMT is involved in neural crest development; progenitor proliferation is associated with stem cell maturation and commitment while fertilization; zygote formation and migration of the blastocyst to the uterine wall involves cell migration. In the context of cancer, EMT is involved in metastasis, progenitor proliferation, increased self-renewal and immune evasion while cell migration occurs when cancer cells migrate from organs/ blood vessels to the surrounding tissues. Examples of key transcription factors that orchestrate physiological processes in both embryonic development and cancer are included alongside.

Similarly, Myc is also known as a tumor initiation and maintenance factor. In vitro, *Myc* knockdown in cancer cell lines decreased cell proliferation and, in some cases, induced apoptosis [15,16,17]. Myc overexpression in an embryonic liver leads to proliferation, whereas in an adult liver, it leads to polyploidy and cell growth [18]. These results have been reported in a large number of tumors including epithelial tumors (hepatocellular, breast, squamous carcinoma), hematopoietic tumors (T- and B-cell lymphoma and leukemia) and mesenchymal tumors (osteogenic sarcoma) [19,20,21,22] (Figure 1).

### 1.2. Epithelial-to-Mesenchymal Transition: in Development and Cancer 

Epithelial-to-Mesenchymal Transition (EMT) refers to the fate transformation of a cell from a stable, stationary epithelial cell to a more migratory mesenchymal cell that is resistant to apoptosis [23,24]. This process is as important in implantation, organ development, and embryo formation as in neoplastic transformation [25]. Placental formation, the initiation of the primitive streak, and gastrulation leading to the separation of three germinal layers all involve EMT [26]. Wnt-signaling is particularly important for EMT during these processes—Wnt3 for EMT during gastrulation and Wnt8c for the formation of the primitive streak [27]. Wnt molecules interact with other pathways such as TGF- and FGF receptors in order to regulate EMT during such developmental processes. The development of one of the best studied migratory cells in the embryo, neural crest cells, also requires EMT from the neuroectoderm [28]. These cells travel to different parts of the embryo and contribute to facial musculature and melanocytes for skin pigmentation, among others. Along with signaling through Wnt, Bone Morphogenetic Proteins (BMPs), Fibroblast Growth Factor (FGF), and c-Myb pathways, neural crest cells need to downregulate E-cadherin and N-cadherin expression for migration [29,30,31,32,33,34].

Multiple cancers are marked by the overproliferation of epithelial cells and angiogenesis followed by invasion through the basement membrane [35]. Malignancy is the final stage of cancer cell migration to distant sites. EMT has been shown to be a critical mechanism for the spread of epithelial malignancies. The expression of mesenchymal markers, as is the case with neural crest cells, is a hallmark of EMT in cancer [35]. A spectrum of signaling mechanisms involved in development are implicated in carcinomas. TFs such as Snail, Slug, Twist, and FoxD3 are essential for EMT both in development and cancer progression. Wnt, bone morphogenetic proteins (BMPs), and fibroblast growth factor (FGF) signaling, along with the loss of E-cadherin by epithelial cells, makes physiological processes in embryogenesis and carcinoma metastasis nearly identical [36,37] (Figure 1). 

While during embryogenesis, the remodeling and diversification of tissues proceeds to generate a fully functional organism, mutations in the DNA facilitate EMT in cancer and lead to invasion and metastasis [38]. 

### 1.3. Cell Migration: Essential for Development and Cancer Progression 

Placental or blastocyst cells invading the uterine endometrium and cancer cells invading the juxtaposed epithelial or endothelial cells use similar cellular mechanisms. It is a multistep process including apposition, adherence or attachment, and eventually differentiation following invasion. Angiogenesis is a key process established after invasion and inflammation that ultimately provides nutrition to invading cells [39,40]. As expected, non-classical HLA class I antigens are recruited for both embryonic development and cancer leading to the recognition of both these tissues as self [39,41]. In tumors, and during the second trimester of pregnancy, a TH-2 type anti-inflammatory immune response is initiated in order to fuel tumor growth and provide sustenance for pregnancy, respectively [42,43]. FoxP3, a TF necessary and sufficient for suppressing the immune functions in regulatory T-cells [44], is also known to regulate the differentiation of uterine T-cells into regulatory T-cells [45,46,47,48,49]. Infertility is a consequence of the absence, or reduced expression of FoxP3 [50]. Incidentally, *FoxP3* also happens to be a tumor suppressor gene in breast and prostate cancers [51,52]. The misexpression of *FoxP3*, owing to its location on the X-chromosome can lead to carcinogenesis [52]. Morphogens, TFs, epigenetic factors, and their downstream signaling cascades interact within a cell. We will mention how each of these contribute individually to development and cancer progression in the next section. 

A wide range of morphogens such as Wnt, Hedgehog (HH), BMPs, and FGFs are essential for the patterning and development of a range of structures including limbs, the heart, and central nervous system. A reactivation of these pathways has been observed in tumorigenesis and metastasis. For example, the Wnt pathway is necessary for patterning, fate specification and progenitor maintenance [53]. Mutations in the tumor suppressor gene, adenomatous polyposis coli, can lead to colorectal cancer [54]. Ovarian cancers and hepatoblastoma also show -catenin overexpression, which is downstream of Wnt signaling [55,56]. Downstream players at the time of attachment include cell adhesion molecules such as integrins, immunoglobulin-CAMs, selectins, and cadherins. E-cadherins are important for both mammary gland development as well as mammary tumors [57,58]. 

Epigenetic changes are not just markers of embryonic development and cancer, they also play a major role in both hematopoiesis and the progression of hematological cancer. DNA methyltransferases are particularly high in embryos [59] and tumors [60,61]. Ten eleven translocation (TET) enzymes are multi-domain enzymes, important for regulating DNA methylation [62]. They are highly expressed in blastocysts at the time of attachment and invasion, and are essential for the survival of an organism [63,64]. TET enzymes are essential for DNA repair and chromosomal translocations in normal physiological conditions and therefore prevent carcinogenesis. Mutations in these enzymes have been implicated in myeloid leukemia [65] and late onset B-cell lymphoma [66,67]. *TET2* was identified as a tumor suppressor and mutations across the gene have been implicated in acute myeloid leukemia (AML), myeloproliferative neoplasms (MPN), and myelodysplastic syndrome (MDS) [68]. 

TFs are at the heart of influencing any cancer initiation and progression. The LIM-homeodomain TFs, reversion-induced Lim (RIL) or PDZ and LIM domain 4 (PDLIM4), promote apoptosis in cancer cells and, hence, are silenced epigenetically in AML and MDS [69], as well as breast cancer [70]. Earlier reviews have drawn comparisons between development and cancer with a focus on individual TF families such as *GATA* or *Pax* in the context of specific organs [71]. In this review, we introduce four of the many TF families that are essential for the regulation of different aspects of embryonic development as well as cancer; basic Helix-loop-Helix (bHLH), GATA, High Mobility Group box (HMG) and Paired box (Pax) TFs (Table 1). We have chosen these families since they have been extensively studied in both development and disease. Parallels between the role of TFs in development and cancer across multiple TF families, and spanning different organ systems, reveal a broad biological phenomenon, and therefore fundamental to understanding and eventually targeting cancer. We discuss the domain structure of the TFs and their role as regulators of development and cancer. We also highlight their role in development across different species to emphasize their evolutionary conservation. Transcriptional regulation is essential for almost every process in the existence of an organism. We will therefore discuss the role of this regulation in the prevention of cancer progression and conclude our review by discussing some of these molecules as candidates for therapeutics and lessons we can learn from species that are cancer resistant (Figure 1). 

## 2. High Mobility Group Box (HMG) 

The HMG-box domain was originally identified as a duplicated 80-amino acid L-shaped domain which binds the DNA minor groove and nucleosomes and, thereby, can induce structural changes in the chromatin fiber [72,73]. HMGs are the most abundant non-histone ubiquitous chromatin proteins in a cell and can be divided into three structurally distinct classes, namely HMG-nucleosome binding family (HMGN), HMG-AT-hook family (HMGA), and HMG-box family (HMGB) [72,74,75,76,77] (Table 1). 

### 2.1. HMG Proteins: A Superfamily of Chromatin Remodelers

HMGN proteins are characterized by a bipartite nuclear localization signal (NLS), a nucleosome-binding domain (NBD) and an acidic C-terminal [78]. HMGN proteins bind specifically to nucleosome core particles to alter and regulate chromatin structure and function [78]. HMGA proteins contain three copies of a conserved DNA-binding peptide motif called the ‘AT-hook’ and an acidic C-terminal tail [79,80]. The AT-hook motif is positively charged and preferentially binds to the AT-rich sequence in the minor groove of DNA [81]. By binding the DNA, HMGA proteins can induce structural and/or conformational changes in the DNA, as well as promote the recruitment of additional components, most of which are TFs [82]. HMGB proteins are the most abundant in the family, with each mammalian nucleus containing approximately 10^5^ to 10^6^ molecules [83]. They interact with proteins implicated in a diverse range of DNA-dependent cellular processes, including DNA replication, recombination, the maintenance of genome integrity, and transposition, among others [84] (Table 1).

### 2.2. Role in Development

HMGN proteins are expressed in all embryonic tissues and are linked to differentiation [85,86]. *HMGN1*^−/−^ mice are subfertile, hypersensitive to various stress conditions, such as exposure to UV light or ionizing irradiation (IR), and have slight defects in corneal epithelium development and maintenance [87,88,89]. HMGA proteins have a role in differentiation and spermatogenesis [90]. *HMGA1*^−/−^ mice suffer from cardiac hypertrophy, hematological malignancies, and type 2 diabetes [91,92]. *HMGA2*^−/−^ mice are pygmies characterized by reduced fat tissue and craniofacial defects [93,94]. *HMGB* genes are expressed in both embryonic and adult tissue [95]. HMGB1 and B2 proteins are important for neural stem cell proliferation, differentiation, and maintenance [96]. *HMGB1*^−/−^ mice show defects in the activation of the glucocorticoid receptor and die within a day of birth due to hypoglycemia. *HMGB2*^−/−^ mice show defects in spermatogenesis and the maintenance of articular cartilage homeostasis in adults, while *HMGB3*^−/−^ mice exhibit erythrocythemia [97,98,99,100,101,102] (Table 1).

### 2.3. Evolutionary Conservation

The HMG boxes of these proteins are well conserved through evolution with homologs in plants, yeast, flies, worms, mammalian cell lines, and animals [192,193,194]. Despite the conservation of the primary sequence among members of this superfamily, several genetic mechanisms have resulted in structural and functional diversity within members [192,193,194]. Phylogenetic and sequence analyses have revealed three possible mechanisms for this divergence, namely (i) gene duplication from an ancient box, (ii) exon shuffling/intragenic duplications to explain why some members of the family carry several HMG boxes, and (iii) the slow accumulation of mutations in newly duplicated genes [192].

### 2.4. Role in Cancer

Tumor markers are detectable in body fluids such as blood serum and urine, and are powerful tools for cancer detection and prognosis. A change in the transcriptional profile of HMGs has been reported in several cancer types. HMGA1 is overexpressed in colon carcinoma, breast carcinomas, and invasive ovarian carcinomas, whereas it was not detectable in normal colon, breast, or ovarian tissue [106]. HMGA1 and HMGA2 are overexpressed in pancreatic adenocarcinomas and non-small cell lung carcinomas (NSCLC), in both squamous and adenocarcinoma histotypes [106]. *HMGN1* regulates the transcription of proto-oncogenes and pro-metastatic genes like *c-fos*, *BCL3*, *N-cadherin*, *JunB* and *c-Jun* involved in tumor progression, in a way which may suppress the development of cancer [103]. The mRNA and protein expression levels of HMGB1 are increased in the lungs of patients with NSCLC, pancreatic ductal adenocarcinoma (PDAC), gastric cancer, colorectal cancer, hepatocellular carcinoma (HCC), and correlate with disease development, tumor progression, invasion, poor prognosis, and metastasis [107] (Table 1).

## 3. GATA Transcription Factors

The discovery of the GATA TF family has transformed the field of hematology. GATA1, the founding member of the GATA family, was initially described as a TF binding to DNA sites within the regulatory regions of several members of α and β-globin families in chickens. Also known as *Eryf1*, *GATA1* was subsequently cloned, purified, and characterized as a ‘switch factor’ in erythroid development [195,196]. This led to the cloning of other members of the GATA family, *GATA2* to *GATA6*. The GATA family shares two highly conserved C2H2-type zinc-finger motifs (Cys-X2-C-X17-Cys-X2-Cys (ZNI and ZNII)) that are involved in DNA-binding by recognizing the GATA element (A/TGATAA/G) [108]. *GATA1*, along with *GATA2* and *GATA3* are collectively grouped as a hematopoietic GATA subfamily, while *GATA4*, *GATA5*, and *GATA6* are classified as an endodermal GATA subfamily [110,197] (Table 1). 

### 3.1. Role in Development

GATA1 functions by promoting the development of erythrocytes, megakaryocytes, mast cells, and eosinophils [109,198,199]. The loss of *GATA1* leads to a substantial increase in GATA2 expression, indicating that GATA1 not only suppresses *GATA2* transcription during erythropoiesis, but is also partly compensated by GATA2. This phenomenon, also known as the ‘GATA switch’, is facilitated by the displacement of GATA2 from its enhancer by overexpressing GATA1 [111]. GATA3 plays a pivotal role in T-cell lymphopoiesis—from the generation of T-cell progenitors to CD4+ specification. GATA3 has also been shown to regulate the self-renewal and differentiation of long-term hematopoietic stem cells (HSCs) in the bone marrow [115,200,201]. A deficiency of *GATA3* during embryogenesis drastically reduces HSC production in the aorta-gonads-mesonephros region [202]. 

GATA4, 5, and 6 are highly expressed in the mesoderm and endoderm-derived tissues such as the stomach, liver, heart, lung, and gonads. GATA4 induces angiogenic factors such as vascular endothelial growth factor (VEGF) to regulate cardiac angiogenesis by promoting compensation after injury [117]. Cyclin D2 and GATA4 have been shown to interact and form a positive feedback loop that enhances the cardiogenic activity of GATA4 [203]. Furthermore, GATA4 promotes bile absorption in the proximal ileum to restore bile homoeostasis [118]. In the developing heart, GATA5 is expressed in both the myocardium and endocardium of mouse embryos. The deletion of both the isoforms of *GATA5* has been shown to result in hypoplastic hearts and partially penetrant bicuspid valve [121,122]. GATA6 has been demonstrated to play a role in the proper patterning of the aortic arch arteries, liver bud growth, and commitment of the endoderm to a hepatic cell fate [124,204]. GATA6, along with its target gene, *Wnt2*, forms a forward transcriptional loop to control posterior cardiac development [125] (Table 1).

### 3.2. Evolutionary Conservation

GATA transcriptional regulators are widely distributed in fungi, plants, and metazoans and their DNA-binding domain is characterized by the presence of one or more class IV zinc finger motif(s). Fungal GATA factors have been shown to be involved in diverse functions such as nitrogen control, siderophore biosynthesis, light-regulated photomorphogenesis, circadian regulation, and mating-type switching [205]. In vertebrates, the zinc-finger domains are more than 70% conserved among all the six GATA binding proteins, although lower homology exists among their amino- and carboxy-terminal sequences [121]. In non-vertebrates such as *Drosophila melanogaster* and *Caenorhabditis elegans*, GATA TFs contain only a single zinc-finger motif that has undergone modular evolution [206]. Since vertebrates and invertebrates share only one C-terminal zinc finger (ZNII), it is possible that a single tandem duplication event might have occurred before the fungal and metazoan lineages diverged, resulting in two zinc finger motifs in vertebrates [207]. 

Evolutionary analysis reveals that the plant GATA family is much larger, more varied, and complex. In contrast to one or two-zinc finger motifs in vertebrates and invertebrates, phylogenetic analysis reveals the presence of four different classes of zinc fingers in plants. For example, *Arabidopsis thaliana* and rice (*Oryza sativa*) genomes contain 29 and 28 loci respectively that encode putative GATA factors that can be grouped into seven different subfamilies [208]. The GATA subfamily VI in plants consists of a tri-zinc finger protein, which has not been previously reported in eukaryotes. Plant GATA factors, unlike animals and fungi, have also been found to be associated with additional domains, such as CONSTANS, CO-like, and TOC1 (CCT) domain, an acidic domain or a transposase-like domain, involved in light signaling or nitrate-dependent transcriptional pathways [209]. Although it is unclear, multiple models of evolution including gene duplication and exon shuffling may explain the underlying basis of the GATA family expansion in plants. 

### 3.3. Role in Cancer

The loss of expression, overexpression, or mutation of GATA factors have been associated with a multitude of cancers including leukemia, colorectal, lung, and breast cancers. Acute megakaryoblastic leukemia (DS-AMKL) seen in Down Syndrome patients is mostly associated with point mutations within the N-terminal zinc finger motif of GATA1. This results in a truncated form of GATA1 (GATA1s) that lacks N-terminal amino acids [110]. The presence of this mutation inhibits GATA1’s ability to bind the hematopoietic transcription co-factor FOG1 (friend of GATA) and affects platelet production [210,211]. Although less is known about the direct role of GATA2 in cancer, a subset of human chronic myelogenous leukemia (CML) patients harbor two mutations in the zinc finger domain of GATA2 [112]. Furthermore, GATA2 is required for *Kras*-driven NSCLC tumorigenesis [212]. Nearly 10% of human breast cancers are associated with *GATA3* mutations in the C-terminal zinc finger of ZNII. The downregulation of GATA3 is a strong prognostic marker, especially in the cases of estrogen receptor (ER)-negative breast cancers, and is linked with aggressiveness and poor survival [116,213]. GATA3 restoration in breast cancer cell-lines induces miR-29b expression, leading to repressed metastasis and reduced tumor outgrowth [214,215]. 

The downregulation of *GATA4* and *GATA5* expression due to epigenetic silencing, such as CpG island hypermethylation and histone hypermethylation is often observed in cases of gastric, lung, ovarian, colorectal, oesophageal cancers, glioblastoma, and diffuse large B-cell lymphoma [119]. GATA6 acts as a double-edged sword in different cancer types. For example, it acts a tumor suppressor in astrocytoma while it is overexpressed in human colon cancer and pancreatic carcinoma [126,216,217]. Although not much is known about GATA factors, improved insights into GATA regulation at transcriptional, translational and post-translational levels can be exploited as novel biomarkers in cancer (Table 1).

## 4. Pax Transcription Factors

Paired box (*Pax*) genes encode TFs that orchestrate complex processes such as embryogenesis and are crucial for maintaining stem-cell pluripotency and stem cell-lineage specificity during development [218,219]. Pax proteins are characterized by the presence of three conserved elements: two DNA-binding domains—the paired domain (PD) and homeodomain (HD)—and the short octapeptide sequence (OP) located between the PD and HD domains [220]. Deletion of the OP motif in some Pax proteins is indicative of a transcriptional inhibitory activity [221]. PD is composed of 128 amino acids and makes sequence-specific contacts with DNA. A second paired-type HD domain found in several Pax members consists of 60 highly conserved amino acid residues. It shares strong homology with other homeobox gene products. PD can either bind DNA independently or as a cooperative interaction with HD domain. However, isolated HD domains have not been demonstrated to bind DNA [222,223]. Additionally, a transactivation domain (TD) at the carboxy terminus of Pax is a proline, serine-, and threonine rich region mediating transcriptional regulation [146,224].

The Pax family is composed of nine TFs (PAX1-PAX9) in humans as well as in mice (Pax1-Pax9). They are subdivided into subgroups I–IV based on the presence, absence, or truncation of a homeodomain—subgroup I (PAX1, PAX9), subgroup II (PAX2, PAX5, PAX8), subgroup III (PAX3, PAX7) and subgroup IV (PAX4, PAX6) [225]. The important roles of Pax genes in development underscore their functions in adult tissue regeneration and the repercussions of their aberrant loss, overexpression, or re-expression are associated with pathology (Table 1). 

### 4.1. Role in Development

During development, the temporal and spatial expressions of Pax genes are tightly regulated. Pax expression is observed during proliferation but is switched off during terminal differentiation [223]. Pax gene expression in adult tissues has often been associated with tissue homeostasis. A small fraction of cortical cells in the adult thymus express PAX1, where it promotes the maturation of thymocytes [129]. PAX2 expression has been documented in medullary regions of the adult kidneys, mammary gland, transitional urothelium of the ureter and bladder as well in the epithelial lining of fallopian tubes of females [226,227,228]. Upon kidney injury, Pax2 expression re-emerges and prevents tubular cells from apoptosis in the initial stage of regeneration [131,132]. Pax3, expressed during early neurogenesis, regulates the generation of sensory neurons from precursors that originate from the neural crest [135]. PAX3 is also expressed in muscle stem cells in adults and melanoblasts (melanocyte stem cells) located in the bulge region of hair follicles, where it maintains their undifferentiated state [136]. Although not much is known about Pax4 function, its expression was shown to confer a protective function in pancreatic β-cells, increasing its replicative potential by transcriptionally activating *Myc* expression. It also protects β-cells from apoptosis by the activation of the anti-apoptotic Bcl-xL [140].

PAX5 is involved in B lymphopoiesis, specifically in the pathway regulating V- to -DJ recombination [142]. Intriguingly, re-programming of mature B-cells to pluripotent stem cells was shown to require *Pax5*, in addition to *Sox2*, *Oct4*, *Klf4* and *Myc* [229]. During development, Pax6 is expressed in multiple brain regions and pancreatic islets, and is essential for eye organogenesis [146,230,231]. Pax6 is crucial for neuroectoderm cell fate determination [232]. Furthermore, the delicate balance between neural stem cell self-renewal and neurogenesis is regulated by Pax6 [233]. It was also reported that Pax6 is re-expressed during corneal wound repair. *PAX6* deficiency was correlated with increased stromal cell apoptosis and cell-proliferation [234,235,236]. On the other hand, Pax7 maintains the proliferation and survival of postnatal satellite cells [237]. It is also found in muscle satellite cells, which are needed for tissue repair and regeneration following muscle injury [150]. 

In the adult thyroid, Pax8 plays a role in regulation of thyroglobulin (Tg), thyroid peroxidase (Tpo), and sodium/iodide symporter (NIS) that are essential for thyroid hormone synthesis [151,152,238]. Pax8 is also important for the maintenance of adult thyroid stem/progenitor cells [239]. Additionally, PAX8 expression has been documented in adult kidneys, specifically in the Bowman’s capsule and medullary regions, which are sites of renal stem/progenitor cells [152,240,241]. PAX9, like PAX8, is also expressed in the adult thymus and eosphagus [129]. Furthermore, PAX9 is also important in development of permanent teeth [154]. Thus, although Pax expression is relatively rare in adult tissues, this expression may be crucial for the survival of stem cell populations and maintenance of pluripotency (Table 1).

### 4.2. Evolutionary Conservation

Pax genes are specific to the animal lineage and have not been found in unicellular organisms, fungi or plants so far [220]. Four *Pax* genes (*Pax1/9*, *Pax2/5/8*, *Pax3/7*, and *Pax4/6*) have been found in the basal chordates, amphioxus (e.g., *Brachiostoma floridae*) and tunicates (e.g., *Ciona intestinalis*) [220,242]. Phylogenetic analyses indicated that, in the ancestral chordate, a single *Pax* gene of each subfamily was present, which gave rise to the amphioxus *Pax*. Subsequently, two major rounds of whole genome duplications occurred that gave rise to multiple vertebrate *Pax* subfamily copies [220,242,243]. Another partial duplication occurred subsequently, resulting in the nine *Pax* genes in mammals [244,245]. An alternative scenario would be that more *Pax* genes would have arisen after two whole genome duplications and were then lost during the vertebrate evolution [246,247].

Overall, purifying the selection appears to be the main factor responsible for the molecular evolution of the *Pax* family in chordate species. However, there are some indications of potential group-specific changes that are beyond this general pattern [248]. Phylogenetic analysis revealed that *Pax2* and *Pax5* ancestors were most likely involved in a round of complete vertebrate duplication while *Pax8* was the most recent gene to appear by local gene duplication in this family. Lizards and birds have lost *Pax4* and *Pax8* [248]. Accelerated evolutionary rates were suggested for the *Pax4*, *Pax8*, and *Pax7* genes. Thus, the asymmetric evolution of the *Pax* family genes can be associated with the emergence of adaptive novelties in the chordate evolutionary trajectory [248].

Moreover, two other alternative scenarios have been proposed to explain the evolution of *Pax* genes. One scenario assumes that the first *Pax* gene comprised of the PD domain alone (represented by *Pax*A/neuro) while the second *Pax* gene appeared as a result of the fusion of PD with an HD-containing gene [249]. Such capturing events could have happened several times and given rise to diverse primary *Pax* types [250]. The other scenario considers only one capturing event followed by gene duplications giving rise to the distinct *Pax* forms [249,251,252]. In this model, the *PaxA* gene is not assumed to denote the progenitor type, but instead is a remnant form lacking the HD domain.

### 4.3. Role in Cancer

*Pax* genes belonging to subgroups II and III that contain OP and a partial HD are involved in cell motility, cell survival, and self-sufficiency in growth signals, thus favoring tumor progression [253]. Conversely, *Pax* genes in subgroups I and IV that only contain one of these domains are rarely involved in cancer, or are indicators of favorable prognosis in cancers [225]. 

*PAX1* was found to be hypermethylated in cervical cancer tissues [130]. On the other hand, *PAX2* is expressed in ovarian cancer, renal-cell carcinomas (RCC) and in some bladder carcinomas, where it is crucial for tumor survival since *PAX2* regulates the surface protein metallopeptidase, A Disintegrin and metalloproteinase-domain containing protein 10 (ADAM10) [133]. Over 70% of RCC cell-lines bear deletions/mutations in the Von Hippel Lindau (VHL) tumor suppressor gene that, in turn, promotes PAX2 expression in renal tumors [254,255]. In breast cancer, PAX2 was reported to form a complex with the ER and regulate *Erythroblastic Oncogene B2 (ERBB2)*, thus determining the response to tamoxifen [134]. In addition, resistance to apoptosis in Kaposi’s sarcoma is associated with *PAX2* expression [256]. In a majority of alveolar rhabdomyosarcomas (ARMS), *PAX3* has been shown to undergo chromosome rearrangement with *FOXO1/FKHR* [137,138]. The PAX3-FKHR fusion in ARMS is a strong transcriptional regulator and is thought to be a dominant-acting oncoprotein [257]. PAX3 is also expressed in primary melanomas and its expression in sentinel lymph nodes has been considered as a prognostic marker for aggressive tumors with a poor outcome [139]. PAX4 is upregulated in human insulinomas [258] and functions as a survival factor in rat insulinoma cells via Bcl-xL upregulation [141]. 

Most B-cell neoplasms, including B-cell lymphoma demonstrate PAX5 overexpression [143]. However, in HCC, *PAX5* acts as a tumor suppressor by interacting with the p53 signaling pathway [144]. In breast cancer, PAX5 expression enhances epithelial behavior and is associated with better prognosis in patients [145]. In PDAC, Pax6 promotes cancer progression by the activation of the receptor tyrosine kinase, c-met [147]. Conversely, PAX6 expression was observed to suppress the invasiveness of glioblastoma cells by regulating the expression of matrix-metalloproteinase 2. In addition, PAX6 also reduced angiogenesis and increased glioma cell susceptibility to detachment and oxidative stress [148,149,259]. 

Similar to PAX3, albeit less frequently, *PAX7* also undergoes rearrangements with *FOXO1/FKHR* in ARMS [137]. *PAX8* undergoes rearrangements with peroxisome proliferator-activated receptor γ (*PPARγ)* in thyroid adenocarcinomas [260]. PAX8 was also shown to be essential for basal *E2F1* transcription and maintaining the stability of its TF c-factor, Rb, in renal, ovarian, and thyroid cancers [241]. In addition, PAX8 also regulates telomerase in certain glioblastoma cell lines [153]. PAX9 is amplified and has been implicated in promoting the proliferation of lung cancer cells [155]. Oncogene-induced cell-survival in oral squamous cell carcinomas is mediated by PAX9 [156]. Thus, *Pax* genes play a major role in conferring growth and survival advantages to cancer cells by regulating cell plasticity [261] (Table 1). 

## 5. bHLH Transcription Factors 

Basic helix loop helix (bHLH) TFs are named on the basis of their structure, and have two evolutionarily conserved domains, namely the basic domain that binds to the E-box DNA sequences (CANNTG) to regulate transcription and the helix-loop-helix (HLH) domain, important for protein homo- or hetero-dimerization. Post dimerization, they bind to the E-box. The dimerization happens via two alpha-helices connected by a non-conserved loop region [262]. Class I bHLH molecules are expressed quite ubiquitously, whereas Class II molecules are tissue specific [263,264]. One such tissue specific bHLH factor is Twist, which regulates EMT in both development and cancer [159,160,265]. The bHLH TF superfamily is imperative for proper development, including the fate specification and cell differentiation of almost all the tissues of any organism from flies to humans [263]. One example of the same TF playing important roles in development and cancer is Myc. Elevated levels of MYC are seen in 60%–70% of all cancers [266]. These are also bHLH TFs and play an important role normally in cell cycle, differentiation, and angiogenesis (Table 1). 

### 5.1. Role in Development

Proneural bHLH TFs were first identified in *Drosophila* for their ability to confer neural identity to ectodermal tissue. In contrast, vertebrate bHLH genes act after neural identity has been determined. The *Achete-Schute* complex and *Atonal* are two neural-specific bHLH gene families in vertebrates, based on their homology in flies, that play a wide range of roles in development [262,267]. Proneural genes *Neurogenin 1*, *2,* and *Ascl1* are required for neural differentiation in both the peripheral and central nervous systems (CNS) [268,269,270,271,272,273,274,275]. Neurogenin 2 and Ascl1 have in fact been used for neuronal reprogramming due to their ability to specify cell fates based on their target genes [157,276,277,278,279,280]. *Atoh1* of the Atonal family is also important for the differentiation of granule cells of the cerebellum and of inner ear hair cells [165]. The bHLH family of neural specific genes also include *NeuroD1*, *D2* and *D6*, and the Olig family. These are important factors for differentiation to neurons and oligodendrocytes within the CNS. *NeuroD1* is necessary for the differentiation of inner ear sensory neurons, granule cells of the cerebellum and the hippocampus [166,281]. *NeuroD2* and *D6* are necessary for the formation of the corpus callosum, needed to communicate between the two cerebral hemispheres [168]. *Olig1, 2* and *3* are necessary and sufficient for oligodendrocyte differentiation in the neocortex, spinal cord and the cerebellum, respectively [174,282,283]. 

Besides their extensive role in neural development, bHLH TFs have also been well-studied in the development of other structures. Math1, Neurogenin 3, and NeuroD1 play a sequential role in the development of gastrointestinal entero-endocrine cells—specification, segregation to the secretory lineage and differentiation [284,285,286,287]. Hand1 and Hand2 play critical roles in the proliferation, differentiation, and the morphogenesis of embryonic ventricle cardiomyocytes [170,171,288]. Twist1 and Twist2 play a major role in bone formation or osteogenesis. They are important for osteoprogenitor proliferation and differentiation via FGF signaling [157]. Twist1 is expressed in the skeletal mesenchyme and also important for craniofacial development, also via FGF signaling [158,289,290] (Table 1).

### 5.2. Evolutionary Conservation

bHLH is a large family of TFs that control the developmental and physiological processes of eukaryotes, and exist in fungi, plants, and animals [267]. Several TFs of this family are evolutionarily conserved across different species and play a crucial role during development. Orthologs of Nephew of atonal 3 (*Nato 3*), a proneural gene, are conserved across *Drosophila*, *C.elegans*, mice, and humans. They are highly similar in their bHLH domain [291]. The *Hand* gene family is also highly conserved across *Drosophila* and mammals, and is essential for heart and vascular development [292]. 

In yeast, bHLH TFs promote cell cycle control and transcriptional enhancement [293,294]. The bHLH members are the second largest class of plant TFs and play a pivotal role in plant growth and maintenance. Phytohormone signaling cascades impinge on to bHLH TFs for *Arabidopsis* development and defense [295]. SlPRE2, an atypical bHLH member, controls the pigmentation of tomato fruit and the morphology of the plant [296]. 

The bHLH family has expanded in plants and animals following evolutionarily independent events [267]. It is unclear whether bHLH TFs evolved from a single common ancestor or via domain shuffling from an ancestral protein [297]. Genome segment and tandem duplications are thought to have led to bHLH gene family expansion in plants [298,299], whereas studies in animals suggest single-gene duplication [300]. The field is still debating whether bHLH TFs expanded in parallel with the evolution of multicellularity, or with the colonization of land [301,302]. Evolutionary analyses of several land plants, chlorophytes, and red algae suggest that the first plants had minimal bHLH genes, and that all modern plant bHLH proteins descended and evolved via a large number of gene duplications [302,303]. 

### 5.3. Role in Cancer

An innumerable number of bHLH TFs are important for cellular differentiation, cell cycle arrest, and apoptosis. Therefore, it isn’t surprising that they play a major role in tumor growth and progression. *Myc* is a proto-oncogene that is dysregulated in several types of cancer. Copy number variations in *MYC* occur very frequently among other genetic events leading to human cancers, for example, in PDAC [163]. Myc is downstream of multiple important signaling pathways such as PI3K [304], Notch [305], Wnt-APC [306], and KRAS-ERK [307] that are implicated in different types of cancers. More importantly, Myc is responsible for both initiating, as well as maintain the tumor [164,308]. Hes1 and Hey1 positively regulate p53 levels, a tumor suppressor gene [188]. Both these TFs are dysregulated in several different cancers [309]. Twist induces EMT and is activated during tumor progression [159,160,161]. BHLHE40 (DEC1) and DEC2 are important for the regulation of the cell cycle via cyclin D1 and cell death in oral and breast cancer cells [179,181]. Dec1 also leads to EMT in pancreatic cancer cells [310]. The expression of TCF3 (E2A) is enhanced in prostate cancer, thereby promoting tumor progression—it provides resistance to apoptosis in prostate cancer [311]. Hypoxia-inducible factor 2 alpha (HIF-2) aids the progression of neuroblastoma and other cancers in non-hypoxic conditions by recruiting Argonaut 2 [312].

The bHLH TFs have been shown to be downregulated in pancreatic cancer and, in fact, a high-throughput screen has identified small molecules as bHLH activators, which may be used as therapeutic targets [313] (Table 1). 

## 6. Discussion

### 6.1. Transcription Factors—Crucial Proteins for Development and Homeostasis 

In this review we have discussed four families of TFs that have been well studied in both, development and cancer. However, there are a multitude of TFs that have important roles in multiple physiological processes and derangements. In fact, 294 cancer-related TFs have been listed in different resources [314,315]. The LIM family of TFs has been exhaustively studied in development [316] and cancer [317]. A few other TFs that have been studied considerably well include the specificity proteins (Sp) family [318], forkhead box (FOX) family [319,320,321,322], HOX genes [323,324], ETS-domain TFs [325,326,327], steroid reproductive hormone receptors [328,329] and zinc finger ZBTB proteins, with N-terminal BTB/POZ domains [330]. 

While we have limited our review to a subset of TFs, development and cancer are regulated by a number of epigenetic factors and noncoding RNA molecules. They have been discussed at length elsewhere [331]. Recently, these molecules have also been targeted for cancer therapy [314,332]. 

### 6.2. Therapeutic Targeting of Transcription Factor: Need of the Hour

TFs regulate a wide range of biological processes and therefore are essential for maintaining homeostasis. They account for nearly 20% of the identified oncogenes and although promising candidates for targeting cancer [314,315], they were considered undruggable up until this decade [333]. A better understanding of their mechanisms of action and structural interactions with the cognate DNA sequence and protein regulators have led to the discovery of useful drug candidates. Despite this progress, the immense repertoire of downstream targets, threshold of expression in normal versus cancerous tissue, redundancy, and compensation by other TFs, epigenetic modulation [334], and vastly different mutations in the same gene across individuals [335] makes it arduous for TFs to be effectively targeted [314]. 

Targeted genome editing technology mediated by CRISPR shows great promise in both fundamental and clinical research. It has been employed for the increment or attenuation of gene expression more reliably than any other genetic engineering technology [336,337]. Targeting TFs using this approach could be a reasonable therapeutic route since they control the fate of a cell, in normal physiology and in cancer. Catalytically inactive dCas9 can be recruited to specific sites on the DNA, which is particularly useful when fused to TFs. This would allow the activation or repression of certain downstream genes [338,339]. Direct targeting of cancer markers such as MYC has been explored to reduce genetic alterations leading to uncontrolled proliferation and metastasis [340]. CRISPR may prove useful in such targeting. In addition, the CRISPR system has been tested for light-induced spatio-temporal control of gene expression [341]. DNA break caused by CRISPR/Cas9 triggers two mechanisms of DNA repair: non homologous end-joining (NHEJ) and homology-directed repair (HDR). Of these, HDR is high fidelity and therefore allows precise DNA editing [342,343]. A novel CRISPR-barcoding tool utilizing HDR enables identification of mutation such as *p53* mutation in breast cancer cells (MCF7) and even correcting a mutation, for example, ALK-F1174L in Kelly neuroblastoma cells [344]. 

**Figure 2:**
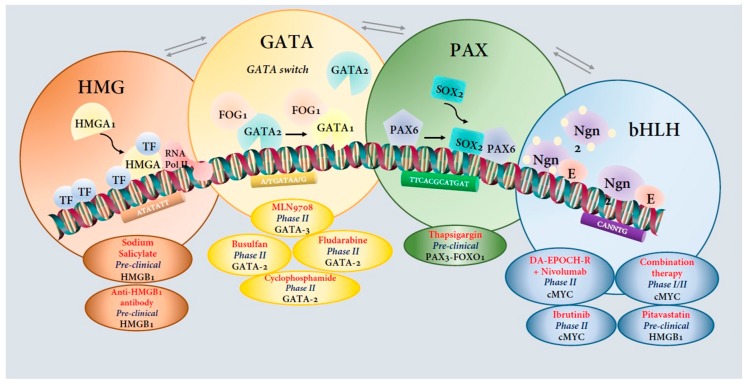
Targeting Transcription Factors in Cancer. Four different Transcription Factor (TF) families, namely HMG, GATA, PAX and bHLH in development and cancer. HMGA1 binds DNA through ‘AT-hook’ motifs to induce or stabilize DNA and/or protein conformations. This triggers enhanced transcription by RNA polymerase II. GATA switch occurs when GATA-1 displaces GATA-2 from FOG-1 when hematopoietic stem/progenitor cells (HSPC) undergo first steps of erythrocytic/megakaryocytic differentiation [349]. PAX6 and Sox2 cooperate functionally and regulate lens development and eye morphogenesis [350]. Two different phosphorylation states of Neurogenin 2 (Ngn2), a bHLH TF, leads to either differentiation or neurogenesis [351]. Examples of candidate drugs targeting each TF are highlighted (Table 2).

As discussed above GATA, HMG, PAX, and bHLH have been implicated in cancer and the characterization of these molecular targets in vitro and in vivo studies have led to the development of several preclinical and clinical studies. The targeted modulation of these TFs can be used for the development of new cancer treatment [337,345,346,347,348]. We have summarized a list of the ongoing preclinical and clinical trials studies for various TF targets (Figure 2, Table 2). 

### 6.3. Natural Resistance Against Cancer: Learning from Life

The task of suppressing somatic mutations in larger organisms and those with a longer lifespan is more challenging. According to Peto’s paradox, there is no correlation between the body size, longevity and increased risk of developing cancer. Therefore, in evolution, larger animals have mechanisms to suppress cancer by either eliminating certain proto-oncogenes or duplicating tumor suppressor genes [358,359,360]. Elephants appear to have low cancer occurrence rates since they have re-functionalized the leukemia inhibitory factor pseudogene 6 (LIF6) with pro-apoptotic functions [361]. In addition, the duplication/multiplication of tumor-suppressor protein TP53 seems to provide another explanation, even though most are processed pseudogenes [362]. DNA damage leads to TP53 upregulation which, in turn, transcriptionally upregulates LIF6. A TP53 response element perhaps evolved co-incident with large body sizes [363]. The analysis of cancer prevention in elephants suggests a lack of understanding of the full extent of the tumor-suppressive capacity of p53 in humans [364]. 

Cetacean species, another order of large mammals could also be effective models for studying cancer [365]. The beluga whales of the St. Lawrence estuary have a high occurrence of cancer, sometimes even surpassing humans, but are an exception among other cetaceans. In pilot whales, bottlenose dolphins, and other toothed whales, cancer is a rare event [366]. The bowhead whales have an extraordinarily long lifespan [367]. Comparative genomics and transcriptomics have revealed the duplication of proliferating cell nuclear antigen (*PCNA*) and other genes involved in DNA repair in these animals [368]. Cross-species comparisons allow us to understand cancer resistance in other mammals as well—for example, naked mole rats and blind mole rats are remarkably resistant to cancer [369].

Fundamentally, plants are different from animals owing to their cell walls. Even though plants develop tumors, the cell wall exerts control on cancer metastasis. Plant tumors are mainly caused by pathogens such as *Agrobacterium* (crown gall), geminivirus, and *Ustinaginales* among other fungal infections [172,370]. In the absence of infections, they are remarkably resistant to neoplastic transformation and hence, cancer. However, spontaneous tumors arise in interspecific hybrids of certain plant species, such as *Nicotiana* (tobacco) [371]. Most of these tumors are caused by phyto-hormonal imbalance [372]. Interestingly, homologs of an extensively studied tumor suppressor and cell cycle regulator, Rb, plays an important role in tumorigenesis in divergent multicellular species [373]. Rb-related (RBR) in plants are implicated in tumor-like growth upon infection with *Agrobacterium* and geminiviruses [374]. Although exhaustive research exists on mammalian Rb and its role in cancer compared to its plant homolog, they have similar roles in cell cycle progression, regulation of TFs via chromatin modifying proteins and role in cell fate decisions [373]. Comparing the molecular aspects of tumor-initiation and progression with plants may provide insights into cancer prevention and the understanding of its biology. 

## Figures and Tables

**Table 1:** The role of transcription factors in embryonic development and cancer.

Transcription Factor Family	Subtype	Role in Development	Role in Cancer
High Mobility Group Proteins (HMG)	*HMGN1*	Corneal epithelium development and maintenance [87,88,89]	Regulates transcription of proto-oncogenes and pro-metastatic genes like *c-fos, BCL3, N-cadherin, JunB and c-Jun* [103]
	*HMGA1*	Regulator of adipogenesis [104], stem cell state [105] and lymphohematopoietic differentiation; crucial for normal sperm production in mouse	Overexpressed in colon, breast and invasive ovarian carcinomas, pancreatic and non-small cell lung adenocarcinomas [106]
	*HMGA2*	Neural crest cells specification in *Xenopus* (essential for animal growth) [93]; governs the exit of embryonic stem cells from pluripotent ground state; cell proliferation and distal epithelium differentiation during embryonic lung development	Overexpressed in pancreatic and non-small cell lung adenocarcinomas [106]
	*HMGB1*	Neural stem cell proliferation, differentiation, and maintenance [96]	Overexpressed in pancreatic (PDAC), gastric, colon, hepatocellular, and non-small cell lung adenocarcinomas [107]
GATA	*GATA1*	Development of erythrocytes, megakaryocytes, mast cells, and eosinophils [108,109]	Mutations in *GATA1* in Down syndrome patients associated with DS-AMKL [110]
	*GATA2*	Hematopoiesis [111]	Mediates Kras-driven tumorigenesis in NSCLC; *GATA2* mutated in a subset of human CML [112,113,114]
	*GATA3*	T-cell lymphopoiesis, self-renewal, and differentiation of long-term HSCs [115]	Tumor suppressor and strong prognostic marker in breast cancer [116]
	*GATA4*	Cardiac angiogenesis and bile homoeostasis [117,118]	Downregulated in gastric, lung, ovarian, colorectal, esophageal, glioblastoma, and large B-cell lymphoma [114,119,120]
	*GATA5*	Cardiac development [121,122]	Downregulated in gastric, lung, ovarian, colorectal, esophageal, glioblastoma, and large B-cell lymphoma [119,123]
	*GATA6*	Hepatic and cardiac development [124,125]	Tumor suppressor in astrocytoma; overexpressed in colon and pancreatic cancer [126,127,128]
PAX	*PAX1*	Maturation of thymocytes [129]	Hypermethylated in cervical cancer [130]
	*PAX2*	Prevention of tubular cells from apoptosis post-injury [131,132]	Overexpressed in ovarian, renal cell, and bladder carcinomas. Regulates *ERBB2* expression in breast cancer [133,134]
	*PAX3*	Early neurogenesis; regulation of sensory neuron generation from precursor cells. Maintenance of undifferentiated state of muscle stem cells [135,136]	PAX3-FKHR fusion protein acts as an oncogene in alveolar rhabdomyosarcomas. Overexpressed in primary melanomas [137,138,139]
	*PAX4*	Protection of pancreaticβ-cells from apoptosis [140]	Upregulated in human insulinomas [141]
	*PAX5*	B lymphopoiesis [142]	Tumor suppressor in hepatocellular carcinomas; overexpressed in B-cell neoplasms; good prognostic marker in breast cancer [143,144,145]
	*PAX6*	Eye organogenesis and neural stem cell self-renewal, neuroectoderm cell fate determination [146]	Oncogenic role in pancreatic adenocarcinoma and glioblastoma [147,148,149]
	*PAX7*	Proliferation and maintenance of postnatal and muscle satellite cells [150]	PAX7-FKHR fusion protein acts as an oncogene in alveolar rhabdomyosarcomas [137]
	*PAX8*	Thyroglobulin regulation; maintenance of thyroid progenitor cells [151,152]	Oncogenic role in renal, ovarian, lung, and thyroid cancers and certain glioblastoma subtypes [153]
	*PAX9*	Development of permanent teeth [154]	Oncogenic role in lung cancer and oral squamous cell carcinomas [155,156]
bHLH	*TWIST 1*	Osteogenesis and craniofacial development [157,158]	Induces EMT; activated during tumor progression [159,160,161]
	*TWIST 2*	Osteogenesis and bone proliferation [157]	Induces EMT; activated during tumor progression [159]
	*MYC*	Skeletal development, osteogenesis, stem and progenitor cell maintenance and self-renewal, organogenesis [162]	Oncogenic role in various cancer signaling pathways; tumor maintenance; copy number variations observed in pancreatic ductal adenocarcinoma [163,164]
	*ATOH1*	Differentiation of granule cells of the cerebellum and inner ear hair cells [165]	Tumor suppressor; silenced in most colorectal cancers; induces differentiation of gastric cancer stem cells; drives metastasis of medulloblastoma; lineage-dependency oncogene in Merkel cell carcinoma.
	*NEUROD1*	Differentiation of inner ear sensory neurons, cerebellum, and the hippocampus [166]	Survival and migration of neuroendocrine lung carcinomas; cell motility and tumor formation of neuroblastoma; in cooperation with *Otx2*, controls Group 3 medulloblastoma active enhancer landscape [167]
	*NEUROD2*	Formation of corpus callosum, essential for communication between the two cerebral hemispheres [168]	Tumor suppressor and prognostic biomarker in Glioblastoma; copy number gains of *NEUROD2* in male breast cancer (prognostic value) [169]
	*HAND1*	Proliferation, differentiation, and morphogenesis of embryonic ventricle cardiomyocytes [170,171]	Downregulated in medulloblastoma; facilitates proliferation and metastasis in gastrointestinal stromal tumor; silenced in over 90% of human primary colorectal tumors. Methylation of *HAND1* associated with poor survival in gastric cancer; involved in thyroid carcinogenesis [172]
	*HAND2*	Proliferation, differentiation, and morphogenesis of embryonic ventricle cardiomyocytes [170,171]	Tumor suppressor in endometroid endometrial carcinoma. *HAND2* suppression upregulates Fgfs in endometriosis [173].
	*OLIG1*	Oligodendrocyte differentiation in the neocortex [174]	Aberrant DNA methylation in non-small cell lung cancer [175]
	*OLIG2*	Oligodendrocyte differentiation in the spinal cord [174]	Universally expressed in gliomas [176]
	*DEC1*	Embryonic endochondral bone development [177]; upregulated in growth plate cartilage and chondrocytes; cartilage terminal differentiation; blocks myogenesis in bovine cells [178]	Critical in cell cycle regulation and cell death in breast and oral cancer; *DEC1* induces EMT in pancreatic cancer [179]
	*DEC2*	Proliferation and differentiation of chondrocytes; neuronal differentiation; adipogenesis. Negative regulator of proliferation and differentiation of chondrocyte-lineage committed mesenchymal stem cells [180]	Critical in cell cycle regulation and cell death in breast and oral cancer [181]
	*HES1*	Cell fate determination and epidermal development [182]; epidermal development [183]; heterogenous ES cell differentiation [184]; proneural gene expression and neuronal differentiation [185]; brain morphogenesis [186]; development of the arterial pole of the heart; thyroid gland development [187].	Deregulated in several cancers and positively regulate levels of the tumor suppressor gene *p53* [188]
	*HEY1 & HEY2*	Embryonic vascular development [189]; maintenance of neural precursor cells; spatial-temporal pattern of mammalian auditory hair cell differentiation [190]. *HEY1* is involved in odontogenic/osteogenic differentiation and cardiac development [191].	Deregulated in several cancers and positively regulates levels of the tumor suppressor gene *p53* [188]

**Table 2:** Ongoing preclinical and clinical trials on transcription factor targets in different types of cancer.

Molecular Target	Candidate Drug	Condition or Disease	Stage of Testing	Other Targets & Disease Conditions	Direct or Nonselective Inhibition	Reference/ClinicalTrial.gov Identifier
HMGB1	Sodium salicylate	Lung adenocarcinoma	Preclinical	**Targets**—Mitogen-activated protein kinases (MAPK), Caspase 3, NF-κb, p38 kinase, AP-1 **Disease**—Acute Myeloid Leukemia	Nonselective	[352]
HMGB1	Anti-HMGB1 antibody	Colorectal cancer	Preclinical	**Disease**—Stroke, Epilepsy, Neudegenerative diseases, neuropathic pain	Direct	[353]
GATA-3	MLN9708	Lymphoma	Phase II	**Targets**—p38 kinase, Janus Kinase (JNK), NF-κb **Disease**—Breast Cancer	Nonselective	NCT02158975
GATA-2	Busulfan, Fludarabine, Busulfan and Cyclophosphamide	Myelodysplastic Syndromes	Phase II	**Disease**—Chronic Myelogenous Leukemia, Lymphomas	Chemo-therapy	NCT01861106
Pax3-Foxo1	Thapsigargin	Alveolar Rhabdomyosarcoma	Preclinical	**Targets**—Sarco/endoplasmic reticulum Ca^2+^ ATPase (SERCA), Nicotinic acetylcholine receptors	Nonselective	[354]
bHLH	Pitavastatin	Pancreatic cancer	Preclinical	**Targets**—3-hydroxy-3-methyl glutaryl coenzyme A reductase **Disease**—Hypercholestrolemia and dyslipidemia	Nonselective	[313]
Reverse the association between Myc and its obligate bHLH heterodimerization partner, Max	10058-F4	Promyelocytic leukemia	Preclinical	**Targets**—MYCN, Myc/Max dimerization **Disease**—*MYCN*-amplified neuroblastoma, Acute Myeloid Leukemia	Direct	[355]
Myc	Mycro1, Mycro2 and Mycro3	Leukemia	Preclinical	**Targets**—Myc/Max dimerization	Direct	[314,356,357]
MYC	Lenalidomide and Combination chemotherapy	B-cell lymphoma	Phase I/II	**Targets**—CRL4 E3 Ubiquitin ligase **Disease**—Multiple Myeloma	Nonselective	NCT02213913
MYC	Ibrutinib	Gastrooesophageal Cancer	Phase II	**Targets**—Bruton’s tyrosine kinase (BTK), CD20 **Disease**—B-cell cancers such as mantle cell lymphoma, chronic lymphocytic leukemia and Waldenstrom’s macroglobulinemia	Nonselective	NCT02884453
MYC	DA-EPOCH-R followed by Nivolumab	B-cell lymphoma	Phase II	**Targets** (Nivolumab)—PD-L1 **Disease**—Squamous non-small cell lung cancer, renal-cell carcinoma, small cell lung cancer	Nonselective	NCT03620578

## Abbreviations

Acute megakaryoblastic leukemia	AMKL
Alveolar rhabdomyosarcomas	ARMS
basic Helix-loop-Helix	bHLH
Bone Morphogenetic Protein	BMP
Central nervous system	CNS
Chronic myelogenous leukemia	CML
Cystein-rich Polycomb-like Proteins	CPP
Epithelial-to-mesenchymal transition	EMT
Fibroblast Growth Factor	FGF
Forkhead box	FOX
Hedgehog	HH
Hepatocellular carcinoma	HCC
Hematopoietic stem cells	HSCs
Hematopoietic stem/progenitor cells	HSPC
High Mobility Group box	HMG
HMG-AT-hook family	HMGA
HMG-box family	HMGB
HMG-nucleosome binding family	HMGBN
Homeodomain	HD
Homology-directed repair	HDR
Leukemia Inhibitory Factor pseudogene 6	LIF6
Myelodysplastic syndrome	MDS
Myeloproliferative neoplasms	MPN
Nephew of atonal 3	Nato 3
Neurogenin2	Ngn2
Non homologous end-joining	NHEJ
Non-small cell lung carcinomas	NSCLC
Nuclear localization signal	NLS
Nucleosome-binding domain	NBD
Octamer binding transcription factor 4	Oct-04
Octopeptide	OP
Paired box genes	PAX
Paired domain	PD
Pancreatic ductal adenocarcinoma	PDAC
Proliferating cell nuclear antigen	PCNA
Renal-cell carcinomas	RCC
Specificity proteins	Sp
Retinoblastoma	Rb
Ten Eleven Translocation	TET
Transactivation domain	TD
Tumor initiating cells	TIC
Thyroglobulin	Ty
Thyroid peroxidase	Tpo
Transcription Factors	TFs
